# Factors Associated with Family Physician Follow-up 30 Days Post-discharge from a Local Canadian Community Emergency Department

**DOI:** 10.7759/cureus.7008

**Published:** 2020-02-16

**Authors:** Kelly Lien, Barrett A Grattan, Alexandra L Reynard, Jocelynn Peters, Jennifer L Parr

**Affiliations:** 1 Family Medicine, Schulich School of Medicine & Dentistry, Western University, London, CAN

**Keywords:** emergency department, follow-up, discharge instructions

## Abstract

Introduction

Close outpatient follow-up with a specialist or family physician post-discharge from the emergency department (ED) has been shown to increase adherence to antihypertensive medications, decrease mortality in heart failure, and reduce the odds of myocardial infarction or death after ED presentation for chest pain. A Canadian study demonstrated that 21% of patients who left the ED with a new diagnosis of atrial fibrillation, heart failure, or hypertension were not seen by a physician within 30 days. There is a paucity of research investigating why this follow-up does not occur. This study aimed to elucidate factors that are associated with outpatient follow-up by a family physician clinic following discharge from a local Canadian community emergency department.

Methods

A retrospective chart review of patients rostered to a family physician who presented to the community ED in the past two years was conducted. The primary outcome examined was a documented follow-up visit with any physician at the clinic within 30 days of the index ED visit. Patients aged 18 or older at the time of the initial ED visit were eligible for inclusion in the study. Exclusion criteria were the following: patients aged 17 or younger at the time of the initial ED visit, those who were not fully assessed at ED visit (i.e., left against medical advice), those whose charts corresponding to the ED visit were unable to be found, patients who were admitted to any facility within 30 days of ED visit, and patients who died within 30 days of the ED visit. Variables of interest extracted from the ED chart and clinic electronic medical record were the following: Canadian Triage and Acuity Scale (CTAS) score, documented discharge instructions, age, sex, primary residence distance from the clinic, last documented clinic visit before ED visit, and the date of and presenting complaint of the next clinic visit after the ED visit. Data were collected as continuous and categorical variables. Descriptive statistics were used to show the number and percentages of patients who followed up in clinic. Binomial regression analysis was used to determine if a specific variable was associated with patient follow-up. Inter-rater reliability between data abstractors was calculated using Fleiss Κ. An alpha-value of 0.05 was chosen, and SPSS version 25.0 (IBM Corp., Armonk, NY) was used for all statistical analyses.

Results

A total of 234 patients out of 1292 patients met inclusion criteria. 53% of patients were female, and the mean age was 50. Seventy-two (31%) received discharge instructions from the ED physician to follow up with their family doctor. In total, 93 of the 234 patients proceeded to have a documented clinic visit within 30 days (40%). 52% (n = 48) of these were women. Receiving specific discharge instructions increased the adjusted odds of follow-up (OR 3.07, 95% CI: 1.64-5.76; P < 0.05). Patients who followed up also tended to have been seen in clinic in the last three months, but this was not statistically significant.

Conclusion

Receiving specific discharge instructions to follow-up increased the odds that patients followed up with their family physician after discharge from the ED. ED physicians may consider giving explicit instructions to patients to improve monitoring of ongoing clinical issues. More research needs to be conducted on how to improve transitions of care. Countries with different healthcare models may have other barriers to appropriate follow-up.

## Introduction

Outpatient follow-up with a specialist or family physician post-discharge from the emergency department (ED) has demonstrated positive outcomes in a variety of clinical situations, including antibiotic compliance, mortality in heart failure, adherence to antihypertensive medications, and myocardial infarction or death after presentation for chest pain [1-5].

Furthermore, as healthcare costs and ED overcrowding increase, there has been much interest in improving continuity of care from the acute ED to the outpatient setting through modalities such as text messaging, telephone reminders, and scheduling of the proposed appointment before ED discharge [6-8]. The goal is to prevent repeat visits to the ED by having family physicians follow-up on issues addressed by the initial ED visit.

Previous work has explored follow-up compliance and its predictors [9]. For example, American studies found an association between insurance type (e.g., private, Medicare/Medicaid, none) and lack of follow-up [10-12]. Thus, it would be reasonable to assume that in Canada, the universal healthcare should theoretically remove insurance as a variable hindering follow-up. One Canadian study specifically evaluated follow-up care following a new diagnosis of atrial fibrillation, heart failure, and hypertension in the ED. It was found that 21% of patients were not seen within 30 days by a family physician, cardiologist or internist, and only 19.6% were seen by their family physician and a specialist within the same timeframe. Factors associated with poor follow-up included lacking a family physician, comorbid conditions (e.g., renal failure, dementia, stroke, etc.), rural residency, and low socioeconomic status [13]. Likewise, another Canadian study investigated patients presenting with chest pain who were discharged from the ED. 25.1% of these patients were not seen by any physician within 30 days. Like the previous study, patients with medical comorbidities and cardiac conditions were less likely to have follow-up [14].

This study aimed to elucidate factors that are associated with outpatient follow-up by a family physician clinic following discharge from a local community emergency department. Anecdotal evidence from clinic physicians suggests that they are not informed of emergency department visits by their patients until the ED charts are processed and brought physically to their clinic. Thus, we hypothesized that an important factor influencing follow-up would be the emergency physician instructing patients to do so, as they have the medical training to recognise a potential serious health issue requiring further investigation.

## Materials and methods

This study was a retrospective chart review of patients who visited a local ED within the study period of November 1, 2016 to January 1, 2019 rostered to a family physician (JLP) within Southwestern Ontario. Research ethics approval was obtained from the Western University Health Sciences Research Ethics Board in London, Ontario, and the research committee at the community hospital.

The family physician provides comprehensive care within a family health network (FHN). The FHN consists of five staff physicians and their resident learners. Each physician has their own personal roster of patients, but patients who require an urgent appointment may be seen by any physician at the clinic. The FHN services a catchment area with a large Indigenous patient population. The community ED is located approximately 20 minutes (16.4 km) away from the clinic. It was chosen as it is physically the closest ED to the clinic, and three of the five staff physicians also work shifts there as part of their family practice. The primary outcome examined was a documented follow-up visit with any physician at the clinic within 30 days of the index ED visit. Thirty days was chosen as an appropriate time frame for follow-up, as the clinic has same-day triage appointments and multiple providers who can see any patient associated with the clinic. In addition, previous similar studies used 30 days as their follow-up time range. If a patient visited the ED more than once in the study time frame, the most recent visit was taken as the index event. Patients aged 18 or older at the time of the initial ED visit were eligible for inclusion in the study.

Exclusion criteria were the following: patients aged 17 or younger at the time of the initial ED visit, those who were not fully assessed at ED visit (i.e., left against medical advice), those whose charts corresponding to the ED visit were unable to be found, patients who were admitted to any facility within 30 days of ED visit (e.g., retirement home, nursing home, psychiatric facility or hospital), and patients who died within 30 days of the ED visit.

Variables of interest extracted from the ED chart and clinic electronic medical record were the following: Canadian Triage and Acuity Scale (CTAS) score, documented discharge instructions, age, sex, primary residence distance from the clinic, last documented clinic visit before ED visit, and the date of and presenting complaint of the next clinic visit after the ED visit. As a proxy for comorbidity severity, previous medical diagnoses were extracted to calculate a Charlson comorbidity index (CCI), which predicts 10-year survival in patients with multiple comorbidities [15]. Information regarding referral to specialists and any follow-up with a consultant was also collected.

Co-investigators (BG, ALR, JP) were trained by KL using a standardized data abstraction sheet. Each investigator extracted the first 25 patients charts independently. Using these data, inter-rater reliability was assessed to guide abstraction from the remaining charts.

Data were collected as continuous and categorical variables. Descriptive statistics were used to show the number and percentages of patients who followed up in clinic. Binomial regression analysis was used to determine if a specific variable was associated with patient follow-up. Inter-rater reliability between data abstractors was calculated using Fleiss Κ. An alpha-value of 0.05 was chosen, and SPSS version 25.0 (IBM Corp., Armonk, NY) was used for all statistical analyses.

## Results

Of a possible sample size of 1292 patients, 234 met the inclusion criteria for the study. All emergency department charts corresponding to the index visits were accessed. Inter-rater reliability results for the following variables were at 100% agreement with Fleiss Κ = 1.0 (p < 0.05): CTAS score, age, sex, distance from clinic, last documented clinic visit before ED visit, and the date of the next clinic visit after the ED visit. Documented discharge instructions, Charlson comorbidity index, and the reason for the next clinic visit following the ED index visit had Fleiss K of 0.98 (p < 0.05).

The mean age of the patients was 50, with women comprising 53%. Most of the patients lived within 20 km of the clinic (Table 1). The average Charlson comorbidity index was 2. For emergency department visits, the mean CTAS score was 3, and 22 (9.4%) arrived by ambulance. Most patients were seen by non-clinic physicians. Seventy-two (30.8%) patients charts had written documented instructions for the patient to follow up with their family physician (Table 2). However, many of these charts did not have a specific time frame for when to book an appointment.

**Table 1:** Characteristics of patients with a visit to a single local community emergency department during the study time frame ED: Emergency department; SD: Standard deviation.

Characteristic	N = 234
Age (years), mean (range)	50 (18-95)
Sex, count (percent)	
Female	123 (53)
Male	111 (47)
Distance from clinic (km), mean (percent)	
<10	99 (42.3)
10-20	109 (46.6)
20-30	13 (5.6)
30-40	6 (2.6)
40-50	3 (1.3)
50+	4 (1.7)
Charlson comorbidity index, mean (SD)	2 (2.13)
Days since last clinic visit prior to ED encounter, mean (range)	167 (0-4948)

**Table 2:** Emergency department visit characteristics of patients with a visit to a single local community emergency department during the study time frame CTAS: Canadian Triage and Acuity Scale; ED: Emergency department; SD: Standard deviation.

Characteristic	
CTAS score, mean (SD)	3 (0.76)
Method of arrival, count (percent)	
Self	212 (90.6)
Ambulance	22 (9.4)
ED physician	
Clinic physician	21 (9)
Non-clinic physician	213 (91)
Documented discharge instructions to follow up with family physician, count (percent)	72 (30.8)

A total of 93 patients were seen at the clinic within 30 days of their ED visit (Table 3). The reason for their visit was related to the presenting complaint in the ED in 82 patients (92%). Of these, 100% (n = 41) of the patients who were told to follow up discussed their ED concern in their clinic visit. There were no significant differences between the age or sex of patients who followed up. The CTAS score tended to be lower (i.e., patients with a higher acuity) in those who followed up. Other factors that showed a trend toward increasing the odds of following up included the ED physician being a clinic physician and having a recent clinic visit prior to the ED encounter; however, neither of these reached statistical significance.

**Table 3:** Characteristics of patients who did and did not receive follow-up care within 30 days of emergency department discharge CI: Confidence interval; CTAS: Canadian Triage and Acuity Scale; ED: Emergency department; SD: Standard deviation Values in bold are statistically significant (p < 0.05).

Characteristic	Followed-up (93, 39.7%)	No follow-up (141, 60.3%)	Odds ratio [95% CI]
Age (years), mean (SD)	53 (19.4)	48 (19.4)	0.984 [0.955–1.014]
Sex			F vs. M 0.767 [0.419–1.404]
Female	48	75	
Male	45	66	
Charlson comorbidity index, mean (SD)	2.18 (2.04)	1.67 (2.2)	2 vs. 0 5.545 [1.641–18.734] 3 vs. 0 6.962 [1.932–25.088]
Clinic visit within 90 days prior to ED encounter	59 (63%)	72 (51%)	1.327 [0.711–2.478]
CTAS score, mean (SD)	3.09 (0.75)	3.33 (0.73)	-
ED physician as a clinic physician	10 (10.8%)	11 (7.8%)	1.128 [0.433–2.942]
Documented discharge instructions to follow up with family physician			
Yes	41	31	3.069 [1.636–5.755]
No	52	110	
Presenting clinic complaint related to ED visit			
Yes	82 (92%)	-	-
No	11 (8%)	-	
Specialist follow-up within 30 days	16 (17%)	28 (20%)	-
No physician follow-up in 30 days	-	114 (81%)	-

**Figure 1:**
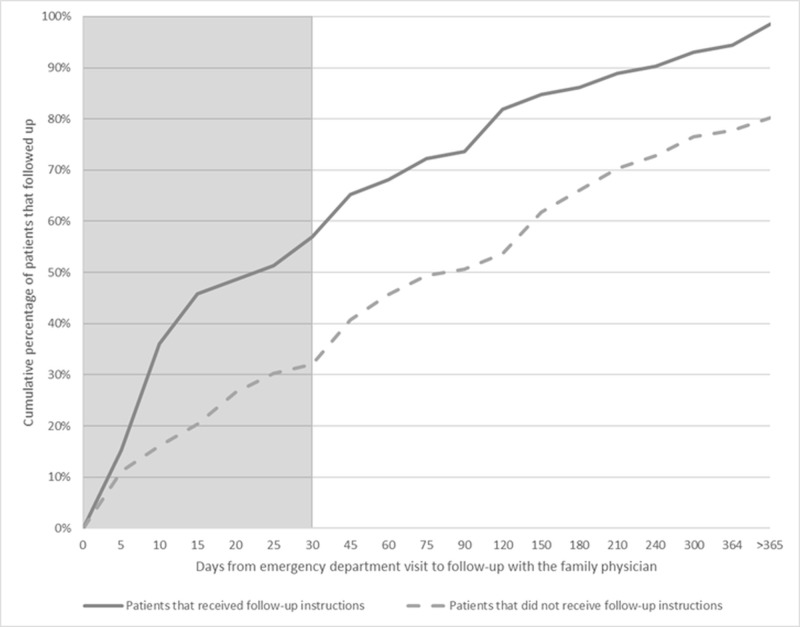
Time course of family physician follow-up after discharge from emergency department. The cumulative percentage of patients who received follow-up within 30 days of discharge from the emergency department is shown by whether patients received follow-up instructions by the emergency department physician.

**Figure 2:**
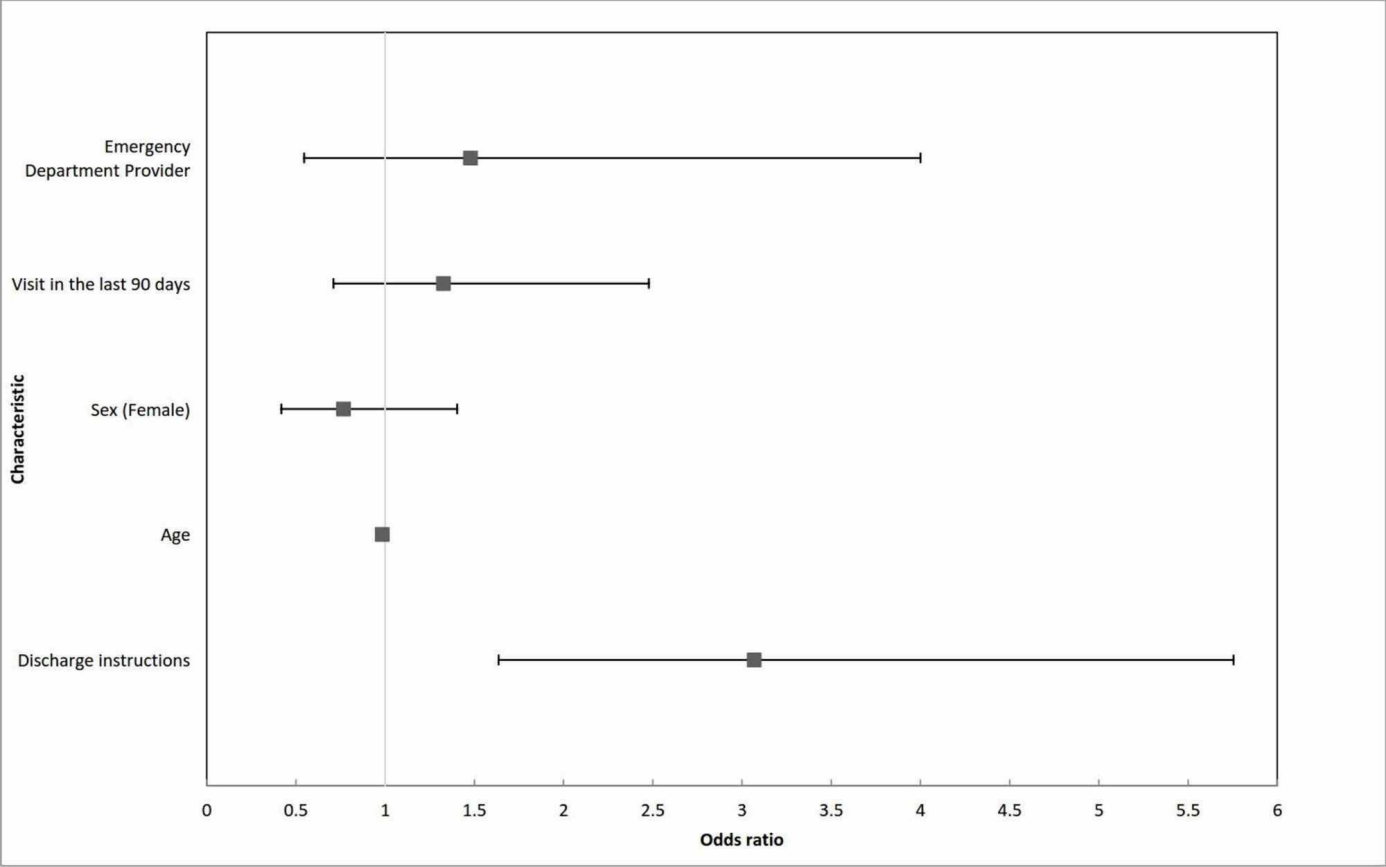
Adjusted odds ratios with 95% confidence intervals for follow-up with the family physician within 30 days of emergency department discharge during the study period. Baseline patient demographics and emergency department characteristics were included in the model.

Patients with a CCI of 2 or 3 were more likely to follow up than those with an index of 0, with higher scores not portending increased odds. The only modifiable factor that increased the odds of follow-up was having documented discharge instructions, which had an odds ratio of 3.069 (P < 0.05). Figure 1 shows the percentage of patients who followed up based on whether they received instructions to see the family physician. By 30 days, 57% of patients (41/72) with instructions were seen in clinic, compared to 32% of patients (52/162) who did not. Specialist follow-up was similar in both groups (17% vs. 20%). Figure 2 shows the odds ratios of selected factors that influence follow-up with 95% confidence intervals.

## Discussion

This retrospective chart review found that the odds of a patient following up with their family physician was increased the most by having documented follow-up instructions in their ED charts. Many of these patients were seen at the clinic within 15 days of their ED visit, and all of them discussed their ED visit at their clinic appointment.

Given that visits to the ED may be the first presentation of a new medical issue or worsening of a chronic one, close monitoring by a patient’s primary care provider is important for continuity of care. In addition, although referrals to specialists may be made from the ED, the family physician plays a crucial role of coordinating care and ensuring any serious health issues are investigated. There is no Ontario-wide integration of health records, so communication regarding ED visits in other cities is extremely limited. Previous research supports this, with most EDs in Ontario still using paper charts that require faxing or mailing to the family physician [16].

With this lack of information sharing between healthcare providers and institutions, it appears that the onus in ensuring follow-up care is on the patients themselves. Our study suggests that this can be improved by the ED physician having a discussion with the patient prior to discharge regarding seeking ongoing care from their family physician. While other studies have tested electronic systems and phone calls to increase follow-up adherence, these interventions require infrastructure, whereas good discharge instructions require only the time of the ED physician. Future research should be conducted to identify which patient populations should have close follow-up. Based on prior studies, this should include patients with multiple comorbidities, lower socioeconomic status, and a rural address [13,14]. In our study, with 90% of the patients living within a 15-minute drive of the clinic, distance was likely not a factor affecting follow-up.

There was a non-statistically significant trend towards follow-up in patients who had been seen by their family physician in the 90 days prior to their ED visit. This could be attributed to the patient being a “regular” at the clinic and having a stronger rapport with the family physician. These results are similar to a previous study, which suggested that a visit to a primary care physician in the previous year was associated with an OR of 6.44 for physician follow-up for patients with chest pain discharged from the ED [14].

There are many reasons why a patient may not have been told to see their family physician after discharge. The presenting ED complaint may have been a simple medical issue that could theoretically be addressed in one visit, such as a urinary tract infection. They may have made a referral to a specialist and assumed follow-up with the primary care physician was not necessary. In these cases, however, it is important that follow-up with the primary care physician is arranged as the wait time for an appointment with a specialist could be in the order of months to years. In addition, the patient should be seen in clinic to inform their physician that a referral was made on their behalf.

Limitations

First, our study is limited to a single community emergency department located 20 minutes away from the family clinic. Following up with their family physician if the patient was seen in an ED in a different city would be logistically difficult in terms of time and transportation. Our study is strengthened by these factors being relatively non-contributory to the odds of following up, but for this reason, our results may not be generalizable to other patient populations. Patients being able to see any physician at the FHN also improves access to care, which may not be possible for family physicians who practice under other compensation models.

Another limitation in our study is that discharge instructions may have been provided by the ED physician that were not recorded in the ED chart. Ideally, these would be documented for medicolegal purposes. We assume that if the instructions were documented, the ED physician spent an adequate amount of time discussing follow-up with the patient.

Nine percent of the patients in our study were seen by ED physicians who also work in the same FHN as the family physician. In a small community, this situation is very common. These physicians may recommend follow-up or not, based on their knowledge of an individual patient’s medical history rather than their presenting complaint. In larger centres where ED physicians do not have family practices (and thus do not have this knowledge), follow-up recommendations may differ.

## Conclusions

To the best of our knowledge, this is the first Canadian study to incorporate specific patient-level data on continuity of care from both the ED and an outpatient family practice clinic. The qualitative data extracted from the electronic medical record (EMR) charts show that patients do discuss their recent ED visits with their family physician, and a provider-level intervention for increasing follow-up is as simple as providing specific instructions prior to discharge from the ED.
